# Family Embeddedness and Medical Students’ Interest for Entrepreneurship as an Alternative Career Choice: Evidence From China

**DOI:** 10.3389/fpsyg.2020.593235

**Published:** 2021-02-11

**Authors:** W. G. Will Zhao, Xiaotong Liu, Hui Zhang

**Affiliations:** ^1^Faculty of Business Administration, Lakehead University, Thunder Bay, ON, Canada; ^2^School of Business, Qingdao University, Qingdao, China

**Keywords:** family embeddedness, entrepreneurial intention, parental attitude formation, medical students, medical school, healthcare

## Abstract

Joining the ongoing academic debates around medical students’ alternative career choices, this research examines the role of family in medical school attendees’ entrepreneurial intention (EI). Specifically, this study decomposes the multidimensionality of family embeddedness and highlights the mediated nature of the family–EI relationship. The empirical analysis relied on data from graduation year medical students from diverse geographical locations and from different institution types in China. These data were collected from a total of 687 questionnaires covering the basic information of individual, parents, and family composition, as well as the measuring scale of EI. Examining medical students’ EI and its antecedents provide a dual-missing-link in the extant knowledge, i.e., it adds the medical school piece to the overall picture of university students’ EI, and equally important, it de-trivializes entrepreneurship from the extant theorizations of medical students’ career choices. This study also bears implications for educators, practitioners, and policymakers interested in better understanding EI of medical school attendees and family embeddedness.

## Introduction

Career choices by medical students have been a tantalizing topic of inquiry for scholars at the intersection of medical education, psychology, and entrepreneurship (Lefevre et al., 2010; Ga̧siorowski et al., 2015). While many may think that medical students typically will become physicians and other healthcare practitioners, there is tremendous evidence that suggests otherwise (Urman and Ehrenfeld, 2011). This highlights the existence of lacunae for a more comprehensive understanding of medical students’ career choices (Griffin and Hu, 2019), especially of factors that influence medical students’ intention to enter entrepreneurship, which has largely been trivialized in the extant research of medical students’ career choice. To contribute to such an understanding, in this study we explore the family factors on medical students’ entrepreneurial intention (EI), drawing on existing insights from entrepreneurship research of family embeddedness (Aldrich and Cliff, 2003; Dyer et al., 2014).

As is established across many disciplines, family is an important context for the passing of the institutionalized way of life in a society (Bourdieu and Johnson, 1993). Research has argued that families and businesses are inextricably intertwined (Aldrich and Cliff, 2003). Family background may have significant implications for one’s entrepreneurial intention and efforts, e.g., providing initial funding (Steier, 2003), mentoring (Sullivan, 2000), information and contacts (Steier, 2007), moral support (Renzulli et al., 2000), and so forth.

Based on empirical data collected from graduating year medical school attendees in China, we offer a more nuanced picture on the family–student EI link in the context of medical school than extant knowledge would allow. We find that medical student’s entrepreneurial attitude and perceived behavioral control, which ultimately impact on their EI, are affected by the attitude of their family (i.e., parents in our case). Meanwhile, said attitudes also vary according to age, income, and profession (especially whether medical students’ parents are physicians). Specifically, younger parents are associated with enhanced interest of their medical school-attending children to pursue entrepreneurship. In terms of household income, parents with the lowest or highest annual household incomes are more likely to support their children to start a business. Medical students from these families exhibit accordingly more interest in entrepreneurship. In addition, medical students whose parents hold managerial positions also show a higher EI. On the contrary, in families where the parents are physicians or other healthcare practitioners, medical students’ EI is lower than the average level. Finally, compared to medical students from the only-child family, those with siblings are more attracted by the entrepreneurial decision.

This article adds to the ongoing debates on EI at the intersection of medical education, psychology, and entrepreneurship in the following ways.

For starters, EI has been a arguably reliable predictor for behavior generation (Krueger et al., 2000; Carr and Sequeira, 2007; Kickul et al., 2008; Liñán et al., 2011; Schmutzler et al., 2019). Examining EI and its antecedents in the medical student context will allow us to provide the medical school piece to the overall picture of university students’ EI (Atitsogbe et al., 2019; Baluku et al., 2020; Karimi and Makreet, 2020; Zhao et al., 2020) and, equally important, to de-trivialize entrepreneurship from the extant research of medical students’ career choice (Lefevre et al., 2010; Ga̧siorowski et al., 2015; Griffin and Hu, 2019).

Second, beyond enhancing the extant knowledge with new context-specific empirical options of capturing family background when analyzing its role on EI, our research advances the family embeddedness perspective of EI *per se* (Aldrich and Cliff, 2003). Despite the noted interests in theorizing family in the context of entrepreneurship (Aldrich and Cliff, 2003) and empirically tackling the family–EI link, e.g., through examining parental interests in entrepreneurship (Luis-Rico et al., 2020), or their interests in getting involved in family business (Zellweger et al., 2011), there is still a paucity of research on the impact of and the mechanisms through which different aspects of family background impacts on offspring’s intention to enter entrepreneurship. Furthermore, despite the long-argued-for importance of family (Renzulli et al., 2000), we still have a rather holistic view on how family impacts on EI, e.g., regarding parents’ entrepreneurial experience and other characteristics as directly affecting offspring’s EI (Tkachev and Kolvereid, 1999; Criaco et al., 2017; Cardella et al., 2020). In this study, more than constituting a valuable supplement to existing research on the direct link between external factors on EI (Peterman and Kennedy, 2003; Souitaris et al., 2007; Kickul et al., 2008; Wu and Wu, 2008; Eesley and Wang, 2017; Wang et al., 2019), we stress the mediated nature of this relationship and de-black-box parents’ attitude formation as a key influencer.

The rest of the paper is organized as follows. We first put forth our family embeddedness perspective. After that, we provide details on the research design and the data. We empirically test our hypotheses on medical students’ EI. Finally, we discuss the implications of our findings.

## Theoretical Orientations

### Furthering the Family Embeddedness Perspective on EI

Existing research argued that the family, through passing resources, norms, and values, has the potential to have a significant impact on entrepreneurship (Aldrich and Cliff, 2003). In this research, we share this perspective and further argue that EI, defined by Liñán et al. (2013) as a conscious awareness and conviction by an individual with the intent to set up a new business venture and plans to do so in the future, is affected by a host of factors collectedly form prospective entrepreneurs’ family influence.

In psychological literatures, intention is often seen as a prerequisite for the actual behavior when it is difficult to observe or when there is an unpredictable time lag. In fact, the decision to become an entrepreneur is a process that takes shape over time (Goethner et al., 2012; Kautonen et al., 2013). As the first step in a series of potential entrepreneurial activities (Krueger et al., 2000), EI therefore is considered to be the simplest and most effective predictor of behavior (Fayolle et al., 2006). Among existing cognitive models on EI, the most notable is the Theory of Planned Behavior (TPB) which has been widely applied in entrepreneurship research various fields (Bird, 1988; Autio et al., 2001; Peterman and Kennedy, 2003; Veciana et al., 2005; van Gelderen et al., 2008; Kibler, 2013; Fayolle and Liñán, 2014; Kautonen et al., 2015; Santos et al., 2016; Feola et al., 2019).

In the TPB model, intention is defined as the willingness of a person to perform a given action, mainly determined by three antecedents, i.e., attitude, subjective norm, and perceived behavioral control. According to this model, subjective norms reflect the influence of external factors on individual decision-making, i.e., the perception that one’s references would or would not approve of one’s decision to take certain actions. Perceived behavioral control, on the other hand, is the ability of an individual to perceive and perform certain behaviors.

In our perspective, individuals are understood as inherently social and susceptible to influence from our surroundings (Cialdini, 2005). In the context of family, family members such as parents are an important source of information, advice, and support for one’s activities (Aldrich and Cliff, 2003). Specifically, as the relationship between parents and children is an essential element of family relationships (Grusec, 2011), we expect that their attitudes would have a positive effect on medical student’s willingness to pursue entrepreneurship as their career choice and that individual entrepreneurial attitude and individual perceived behavior control would have an mediating impact.

H1. There is a positive association between parents’ attitude and medical student’s personal attitude.
H2. There is a positive association between parents’ attitude and medical student’s perceived behavior control.
H3. Personal attitude will have a positive impact on medical student’s EI.
H4. Perceived behavioral control will have a positive impact on medical student’s EI.

### Unpacking Family Embeddedness for Medical School Attendees’ Entrepreneurship Career Choice

Researchers have long argued that human capital, representing the collective knowledge and cognitive abilities of family members (Coleman, 1988), improves the cognitive ability of individuals to successfully build and sustain businesses (Shane, 2003). Research has also shown that age (Kautonen et al., 2011) and education (Martin et al., 2013) are associated with differences in human capital in general. As general human capital comes from life experiences, we expect that medical students’ parents of different age groups and education background may have significantly different attitudes toward the students’ career choice of entrepreneurship. We therefore hypothesize that,

H5. Parents’ age will have a significant impact on their attitudes toward medical student’s choice of entrepreneurship.
H6. Parents’ educational level will have a significant impact on their attitudes toward medical student’s choice of entrepreneurship.

Existing research sheds important light on the importance of entrepreneurial experience (Muñoz-Bullon et al., 2015) as it improves the cognitive skills of family members. Differently put, parents who have prior entrepreneurial experience might think they could help to mobilize their resources needed for successful entrepreneurship. In addition to human capital, scholars also found that people with different financial capitals, such as incomes, evaluate the likely outcome of entrepreneurship differently (Pinillos and Reyes, 2011; Liñán et al., 2013). Given that the availability of financial capital is particularly important for young entrepreneurs (Cassar, 2004), we expect that family financial capital will impact the way family members regard their students’ entrepreneurship. Related to this, as occupational categories are understood as having an impact on medical students’ parents’ expectations on their career choices (Griffin and Hu, 2019), we expect that parents with different types of occupations, such as physicians vs. non-physicians, would have different attitudes on the medical students’ choice of entrepreneurship.

H7. Parents’ prior entrepreneurial experience will have a significant influence on their attitudes to medical student’s choice of entrepreneurship.
H8. Different levels of income will have a significant influence on parents’ attitudes toward medical students’ choice of entrepreneurship.
H9. Parental occupational category will have a significant influence on their attitudes toward medical students’ choice of entrepreneurship.

Finally, given that one-child family is a common feature of Chinese families with increasing exceptions (Deutsch, 2006), to complement the above hypotheses, we are interested to know if medical students’ family compositions affect their parents’ attitudes toward their career choice of entrepreneurship. We therefore hypothesize that,

H10. Whether the family has only one child will have a significant influence on parents’ attitudes toward the student’s choice of entrepreneurship.

## Research Design and Methods

### Data Sources

Previous research on student entrepreneurship typically focuses on final year students (Autio et al., 2001; Veciana et al., 2005; Fayolle et al., 2006). Given our scope of inquiry, in this study, we chose to collect data from final year medical students, not only because they face immediate career choices between becoming a physician or taking on alternative career paths but also to rule out other influences such as postgraduation work experience. To better reflect the overall population of Chinese medical students, we collected data from diverse geographical locations (Eastern, Central, and Western China) and from different institution types (nationally top universities and ordinary ones) in our sample. Specifically, to account for the differences among regions of China in terms of healthcare and education resources, we included three medical schools from Eastern China, two in Central China, and one in Western China. We sent 150 surveys to each institution. The process started from May 2018 and ended in March 2019. A total of 687 questionnaires were collected eventually. 153 of them were removed due to data missing, which renders the total number to 534. The overall sex ratio of the sample was 45.1% for men and 54.9% for women, and the average age was 23.7 years old. Altogether, 104 master students and 25 doctoral students participated in the survey.

### Research Method

The questionnaire contains the basic information of individual and family and the measuring scale of EI. The measurement items of EI and its antecedents (entrepreneurial attitude and perceived behavior control) come from the Entrepreneurial Intention Questionnaire (EIQ) designed by Liñán and Chen (2009). Empirical studies have shown that the scale has good reliability and validity. The measurement of psychological properties for the medical students also shows a good applicability (Wu and Wu, 2008; Liñán and Chen, 2009). The questionnaire utilized six items to measure medical students’ EI, focusing on behavioral aspects of intention (Armitage and Conner, 2001). Perceived behavior control consisted of six measurement items and included both self-efficacy and controllability elements. Personal entrepreneurial attitude was measured using five items employed in prior research (Krueger et al., 2000; Goethner et al., 2012).

We take the perception of parents’ attitude as the main source of subjective norm. We used an aggregate measure for the perceived parents’ attitudes consisting of 4 measurement items, instead of simply using the degree of individual perceived approval from their references (Krueger et al., 2000; Ajzen, 2001; Liñán and Chen, 2009; Liñán and Rodríguez-Cohard, 2015). Table 1 presents the items and descriptive statistics.

**TABLE 1:** Item-construct and descriptive statistics.

**Construct**	**Item**	**Mean**	**SD**
Parental attitude (A)	A1. My parents think that being an entrepreneur may be a good choice for me under the current social environment	4.133	1.418
	A2. My parents respect my choice to start a business out of my own interest	3.845	1.401
	A3. My parents think that young people deserve chances to choose their careers through trial and error	4.331	1.580
	A4. My parents will give me as much support as they can both mentally and financially if I started a business	4.457	1.472
Personal attitude (B)	B1. Being an entrepreneur means more advantages than disadvantages for me	4.493	1.376
	B2. A career as entrepreneur is attractive for me	4.228	1.474
	B3. If I had the opportunity and resources, I would like to start a business	4.493	1.399
	B4. Being an entrepreneur would entail great satisfactions for me	4.401	1.438
	B5. Among various career choices, I would rather be an entrepreneur	4.026	1.480
Perceived behavior	C1. To start a business and keep it working would be easy for me	4.249	1.354
control (C)	C2. I am prepared to start a viable firm	4.189	1.314
	C3. I can control the creation process of a new firm	4.303	1.521
	C4. I know the necessary practical details to start a firm	4.290	1.359
	C5. I know how to develop an entrepreneurial project	4.457	1.486
	C6. If I tried to start a business, I would have a high probability of succeeding	4.318	1.485
Entrepreneurial intention (D)	D1. I am ready to do anything to be an entrepreneur	3.757	1.586
	D2. My professional goal is to become an entrepreneur	3.955	1.554
	D3. I will make every effort to start and run my own business	3.601	1.578
	D4. I am determined to create a business in the future	3.854	1.572
	D5. I have very seriously thought of starting a business	3.772	1.567
	D6. I have strong intention to start a business some day	3.867	1.622

## Parental Attitude Impacting Medical Students’ EI

### Test of Reliability and Validity of Variables

We followed Chandler and Lyon (2001) and used Cronbach’s alpha to test the reliability of the questionnaire. Statistical analyses were carried out using SPSS 24 (IBM). The results show that all values range between 0.83 and 0.929 (see the last row in Table 2), which are all above the widely accepted threshold of 0.7 (Nunnally and Bernstein, 1994). Thus, our measurement scales show good internal consistency and may be considered as reliable. In terms of content validity and structural validity, we took much care to select items for the parents’ attitude construct to ensure the items are both relevant and representative, and we used scales validated in previous studies for the other items.

**TABLE 2:** Factor load matrix after rotation.

	**Factor**			
	**(A)**	**(B)**	**(C)**	**(D)**
A1	0.798			
A2	0.771			
A3	0.773			
A4	0.782			
B1		0.811		
B2		0.783		
B3		0.771		
B4		0.790		
B5		0.759		
C1			0.767	
C2			0.678	
C3			0.709	
C4			0.777	
C5			0.738	
C6			0.739	
D1				0.777
D2				0.648
D3				0.776
D4				0.708
D5				0.726
D6				0.790
Cronbach’s α	0.830	0.929	0.884	0.918

*Extraction method: principal component analysis. Rotation method: Varimax with Kaiser normalization. Rotation converged after six iterations. Loadings below 0.50 not shown.*

Convergent validity in existing research has usually been assessed by factor analysis (Klein and Kleinman, 2002; Kreiser et al., 2002; Klein et al., 2005). In our study, the KMO statistic of the sample is 0.945, which indicates the sample size is sufficient. Also, Bartlett’s sphericity test is also significant (*p* < 0.001), which demonstrates that the strength of the relationship among variables is strong. Thus, it is suitable for factor analysis for the data. Table 2 presents the rotated factor matrix. Four factors were extracted, which is consistent with the questionnaire structure. Moreover, cumulative variance explained by the extraction was 70.114%. As may be observed, each item was restricted to load on its *a priori* specified factor only (all loadings >0.5), which shows that the convergent validity of the measurement scale is ideal.

We also performed a confirmatory factor analysis (CFA) using this structure. The skewness and kurtosis of each item are all between plus and minus 1, which signifies that the sample satisfies the assumption of a normal distribution, and in this case, estimations obtained by maximum likelihood (ML) analysis are precise (West et al., 1995). Therefore, the ML estimator in Mplus 8.3 was employed in this paper to carry out CFA and subsequent structural equation model analysis for sample data. The CFA showed that normalized factor loading values of all observed items were higher than 0.7 and composite reliability (CR) values of four latent variables ranged between 0.831 and 0.929 (>0.7) (Nunnally and Bernstein, 1994), which further reflects preferable internal consistency. Model fit statistics (χ2 = 392.236, df = 183, RMSEA = 0.046, CFI = 0.972, TLI = 0.968, SRMR = 0.030) suggest that the measurement model fits the data reasonably well. To evaluate discriminant validity, the first step is to calculate the average variance extracted (AVE) of any construct and then compare the square root of the AVE with the correlations among the latent variables. As discriminant validity indicates the extent to which a given construct is different from other constructs, the square root of AVE of one latent variable should be greater than the correlation coefficients with other variables (Chin, 1998). Table 3 displays the discriminate validity test results. In line with it, the square roots of all AVEs on the diagonal line are larger than the off-diagonal elements in the corresponding rows and columns, demonstrating good discriminant validity.

**TABLE 3:** Correlation of constructs and square root of AVE values.

**Variable**	**(A)**	**(B)**	**(C)**	**(D)**
Family members’ attitude (A)	**0.743**			
Personal attitude (B)	0.476	**0.851**		
Perceived behavior control (C)	0.473	0.582	**0.750**	
Entrepreneurial intention (D)	0.402	0.755	0.669	**0.810**

*Bold value idicates the square root of each latent variable’s average variance extracted (AVE).*

For common method variance, Harman’s one-factor test was applied to assess whether a method-bias-induced single factor accounted for the covariance in the relationships between independent and dependent variables (Podsakoff and Organ, 1986). After having constrained the factor analysis to only one factor, it appeared that such a factor would account for no more than 50% of the variance, providing initial evidence that common method bias is not a problem because no single factor accounts for most of the variance (Anderson et al., 1998; Hockerts, 2017). As a second precaution against common method bias and to further ensure the validity and distinctiveness of our measures, we estimated different specifications of the CFA model (Podsakoff et al., 2003). First, we compared the fit of a four-factor structure to that of a one-factor structure. As shown above, our four-factor structure fits the data very well. The results of the one-factor structure (χ2 = 2391.246, df = 189, RMSEA = 0.148, CFI = 0.704, TLI = 0.671, SRMR = 0.100) are significantly worse than for the four-factor structure (difference in χ2 = 1999.010, df = 6, *p* < 0.001). We also analyzed two-factor structures and three-factor structures. In every possible specification, the fit of the model was worse than in the original one where all items load on their theoretically specified factors. This indicates that the measures we used are not only theoretically distinguishable but also empirically so and that common method bias should not be a concern in our case (Podsakoff et al., 2003).

### Results

**FIGURE 1:**
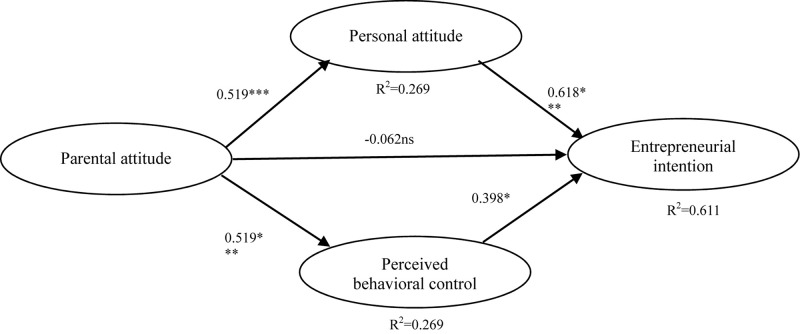
Estimation results for the path analysis. χ2 = 481.804, df = 184, RMSEA = 0.055, CFI = 0.960, TLI = 0.954, SRMR = 0.0089. Significant levels: *p < 0.05; ^∗∗^*p* < 0.01; ^∗∗∗^*p* < 0.001.

**FIGURE 2:**
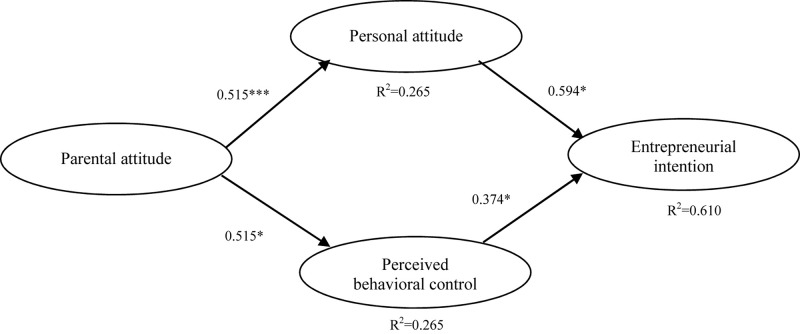
Estimation results for the path analysis. χ2 = 483.709, df = 185, RMSEA = 0.055, CFI = 0.960, TLI = 0.954, SRMR = 0.090. Significant levels: ^∗^*p* < 0.05; ^∗∗^*p* < 0.01; ^∗∗∗^*p* < 0.001.

We assessed the model with the SEM analysis via Mplus 8.3. As shown in Figure 1, only the relationship between parental attitude and EI is not significant—similar to results by other researchers (Krueger et al., 2000; Autio et al., 2001; Liñán and Chen, 2009; Santos et al., 2016). Therefore, we chose a more concise model that removed the direct path of parental attitude on EI. Figure 2 shows all path coefficients and model fit statistics. The estimation results show satisfactory fit indices, except SRMR (χ2 = 483.709, df = 185, RMSEA = 0.055, CFI = 0.960, TLI = 0.954, SRMR = 0.090). Besides, all path coefficients are significant (*p* < 0.001). Hypotheses 1, 2, 3, and 4 are therefore accepted. These results mean that medical students who perceive their parents as thinking more positively about their entrepreneurship career choice will have stronger entrepreneurial attitude and perceived behavioral control. Meanwhile, personal attitude and perceived behavioral control will further exert positive influence on the EI of medical students. Also, the model explains 61.0% of the variance in EI based on entrepreneurial attitude and personal behavior control. This value is even higher than the results of Liñán and Chen’s study on Spanish and Taiwanese university students, which are 57.9% and 57.8%, respectively (Liñán and Chen, 2009). We can therefore consider that the revised model shows better explanatory power for the analysis of Chinese medical students’ EI. The standardized path coefficient and its standard error of the whole model are summarized in Table 4.

**TABLE 4:** Standardized regression weights of entrepreneurial intention model.

**Path**	**Estimate**	**SE**	**Hypotheses**
Parental attitude → A1	0.738***	0.029	
Parental attitude → A2	0.723***	0.030	
Parental attitude → A3	0.746***	0.031	
Parental attitude → A4	0.744***	0.032	
Personal attitude → B1	0.820***	0.018	
Personal attitude → B2	0.870***	0.014	
Personal attitude → B3	0.854***	0.014	
Personal attitude → B4	0.842***	0.014	
Personal attitude → B5	0.870***	0.013	
Perceived behavior control → C1	0.772***	0.025	
Perceived behavior control → C2	0.718***	0.030	
Perceived behavior control → C3	0.739***	0.028	
Perceived behavior control → C4	0.798***	0.024	
Perceived behavior control → C5	0.743***	0.024	
Perceived behavior control → C6	0.731***	0.026	
Entrepreneurial intention→ D1	0.729***	0.028	
Entrepreneurial intention→ D2	0.701***	0.033	
Entrepreneurial intention→ D3	0.758***	0.027	
Entrepreneurial intention→ D4	0.818***	0.025	
Entrepreneurial intention→ D5	0.825***	0.022	
Entrepreneurial intention→ D6	0.924***	0.011	
Parental attitude → Personal attitude	0.515***	0.047	Support
Parental attitude → Perceived behavior control	0.515***	0.046	Support
Personal attitude → EI	0.594***	0.044	Support
Perceived behavior control→ EI	0.374***	0.048	Support

Significant levels: *p < 0.05; **p < 0.01; ***p < 0.001.

Furthermore, we performed path analysis with the bias-corrected percentile bootstrap, bootstrapping 5,000 samples to compute bias-corrected confidence intervals. The results suggest that the indirect relationship between parents’ attitude EI via personal attitude is significant [*b* = 0.306, SE = 0.034, 99.9% confidence interval (CI) = (0.243, 0.396)]. Similarly, the indirect relationship between parents’ attitude and EI via perceived behavior control is also significant [*b* = 0.192, SE = 0.028, 99.9% (CI) = (0.142, 0.256)].

## Influencers of Parental Attitude

To test the factors leading to differences in the parental attitude, we explored the influence mechanism of various relevant factors on parental attitude.

### Variable Definitions and Descriptive Statistics

Dependent Variable: The factor analysis of the four observation items related to parental attitude gave us the score coefficient of each observation indicator, which reflects the important level of each observation indicator to the explanation of the common factor. Therefore, this score coefficient could be adopted as weight. After normalization processing, the comprehensive score of parental attitudes was obtained to be regarded as the dependent variable.

Independent variables: To recap, independent variables include parental age, educational level, occupational category, entrepreneurial experience, family income, and family composition. Age, educational level, occupational category, and entrepreneurial experience are specific information on individual characteristics. Therefore, we mainly focused on the one of parents who has a greater impact on the career choice of respondents in the questionnaire. Due to the diversity of occupations, we adopted an integrated classification approach that considered occupational hierarchy and the nature of employer. This approach gave us six occupational categories: public servant, professionals in business and industries, physician and other healthcare practitioners, professionals in science and education, farmer (including peasant-workers without permanent residence in cities), and other professionals. Parents’ education levels were divided into eight ordinal levels: “1” indicates elementary school dropouts and below, while “8” represents doctorate. The other two classification indicators are for whether parents’ have entrepreneurial experience and whether the medical student is a single child. If the answer was yes, the variable would be set as 1, otherwise the variable would be set as 0. In addition, annual household incomes (RMB) were processed by logarithmic processing, with the addition of the term of squared income, to observe possible non-linear correlations between parents’ attitude and household income. Finally, we also introduced respondents’ traits (gender, age, and stage) as controlling variables. Descriptive statistics for the measurement variables are presented in Table 5.

**TABLE 5:** Descriptive statistics of variables.

**Variable**	**Min**	**Max**	**Mean**	**SD**
Parental attitude	1	7	4.193	1.196
Parents’ age	42	69	48.431	3.236
Parents’ educational level	1	8	3.831	1.718
Parents’ entrepreneurial experience	0	1	0.348	0.477
Income	8.294	13.998	11.033	0.908
Income*Income	68.791	195.939	122.543	19.669
**Occupation**				
Public servant			11.985%	
Professionals in business and industries			16.105%	
Physician and other healthcare practitioners			23.034%	
Professionals in science and education			13.483%	
Farmer			20.787%	
Other professionals			–	
Only child (or not)	0	1	0.590	0.492
Respondent’s gender	0	1	0.451	0.498
Respondent’s age	19	36	23.652	2.379
Respondent’s stage	0	1	0.242	0.428

### Results

The statistical analysis was performed using SPSS 24 (IBM). The results of multiple regression analysis are shown in Table 6.

**TABLE 6:** Multiple regression analysis of parental attitude.

**Variables**	**Model 1**	**Model 2**
***Control***		
Gender (1 = male)	−0.025 (0.104)	0.237* (0.118)
Age	−0.014 (0.033)	0.044 (0.038)
Stage (1 = graduate student)	−0.340 (0.182)	0.195 (0.179)
***Independent***		
Family member’s age		−0.124*** (0.035)
Family member’s educational level		0.068 (0.043)
Family member’s entrepreneurial experience (1 = Have entrepreneurial experience)		0.085 (0.098)
Income		−7.334*** (0.819)
Income*Income		0.333*** (0.037)
Family member’s occupation (Other professionals)		
*Public servant*		−0.013 (0.208)
*Professionals in business and industries*		0.357* (0.176)
*Physician and other healthcare practitioners*		−0.424* (0.173)
*Professionals in science and education*		0.017 (0.208)
*Farmer*		−0.261 (0.178)
Only child or not (1 = the single-child)		−0.402*** (0.102)
Intercept	4.617*** (0.743)	49.110*** (4.578)
*R*^2^	0.021	0.308
Adjusted *R*^2^	0.015	0.289
F	3.797*	16.474***

Dependent variable: Parental attitude. Significant levels: *p < 0.05; **p < 0.01; ***p < 0.001.

These results indicate that age, family income, family composition, and occupational category have a significant impact on parental attitude. Therefore, hypotheses 5, 8, 9, and 10 are verified. Parents’ educational level and prior entrepreneurial experience are not significant at the 95% level. Specifically, parents’ age is negatively associated with their attitudes for medical students’ career choice of entrepreneurship. In our sample, the respondents’ parents are between 42 to 69 years old, which potentially means that older parents exhibit a higher tendency of risk aversion (Kan and Tsai, 2006; Lévesque and Minniti, 2006).

The coefficients of household income and its square term are significant at the 99.9% level (*p* < 0.001), with the annual household income and parents’ attitude basically forming a U-type relationship. That is, with an increase of annual household income, parents’ supportive attitude to medical students’ choice of entrepreneurship decreases first, but then increases. One possible explanation is that low-income families tend to be in the lower social strata, and such families often have limited social resources, making it difficult to help the career development of the students in these families. For high-income families, on the one hand, the financial resources may better enable them to deal with potential entrepreneurial failure. Therefore, these family members are usually willing to respect medical students’ career choices. Also, compared with the middle-income families, higher-income ones generally have more social resources and therefore, medical students from high-income families are more likely to perceive the support of their parents for their entrepreneurial career choices. As far as family composition is concerned, we found that number of children in a family had a significant impact on the parents’ attitude. Parents of an only child tend to have more negative attitudes toward medical students giving up their potential career in healthcare for entrepreneurship.

Our results also show that different occupational categories are associated with differences in parents’ attitudes, with “other professionals” as the control group. While we found no significant difference between categories of the public servant, professionals in science and education, farmers, and the control group, there are two occupational categories that are noteworthy: business-related jobs and healthcare-related ones.

Parents with a job in business and industries tend to be more supportive of children’s entrepreneurial choices; a result, to us, is similarly explainable to the reasons why better-paid parents tend to have more positive attitudes. In line with the resource argument (Clough et al., 2019), parents with managerial roles are expected to have more financial and social resources. They also can provide better human resource-based support for their children. It is therefore expected that the parents of this occupational category show more supportive attitude than the control group.

Perhaps unsurprisingly, parents who are the physicians and other healthcare practitioners show negative attitudes to their children’s career choice of entrepreneurship. We think this is possibly be due to the influence of the profession on one’s cognition. While healthcare practitioner turnover is common (Masselink et al., 2008), giving up the profession is not an easy decision, especially when healthcare practitioners are still among the esteemed occupational group in China. Long-term stable and secure working environment is likely to cause family members in this occupational group to further exclude their children from choosing high-risk entrepreneurial activities. For instance, physicians may hope their children to take a job they are familiar with, and they may be indeed better connected in the system than other family members to help their children’s medical career.

## Conclusion and Future Directions

This article highlights that medical students’ career decision can be significantly affected by their family. Going further than existing research, our findings show that parental attitude formation plays an important role in affecting medical students’ EI, through impact their entrepreneurial attitude and perceived behavioral control. Parents’ attitudes also vary significantly according to their ages, incomes, occupational categories, and family composition.

The first contribution this study makes lies in its attempts to narrow the gap of our knowledge on medical students’ intention to enter entrepreneurship, which has been trivialized in existing research on medical students’ career choices (Lefevre et al., 2010; Ga̧siorowski et al., 2015; Griffin and Hu, 2019). Studying Chinese medical students’ EI also presents an important addition to our existing knowledge on students’ EI (Atitsogbe et al., 2019; Baluku et al., 2020; Karimi and Makreet, 2020; Zhao et al., 2020), especially when Chinese medical students are said to overwhelmingly favor a position in a well-ranked public hospital than practicing independently (She et al., 2008). Further research is needed to expand our findings to other external factors of EI and could be of value to analyses of specific groups’ EI and activities, e.g., nurses (Roggenkamp and White, 1998). Future studies could focus on the uniqueness of the family composition in mainland China and examine how it, together with some unique intergenerational interaction patterns such as Confucian filial piety and the virtue of respects for both father and elders (Yeh et al., 2013), impacts entrepreneurship of medical students in the Eastern context. Future studies could also these findings compare with entrepreneurship interests by medical students in other countries and regions.

Second, our study furthers the family embeddedness perspective of EI. This perspective, originally advocated for the study of entrepreneurship process (Aldrich and Cliff, 2003), is built on the premise that access to certain social structures and members of social groups, such as families, facilitates access to resources that may be useful to entrepreneurs (Granovetter, 1985). Early studies have garnered important insights on factors impacting family embeddedness (Azmat and Fujimoto, 2016). Going beyond the extant knowledge, our study demonstrates the mediated nature of the family–EI link and showcases the importance of attitude formation. Specifically, rather than taking a holistic view on parents’ attitudes, we un-blackboxed parental attitudes and provided a fine-grained understanding on the mechanisms through which parents’ normative and value judgments form the basis of the subjective norms of potential entrepreneurs. In this sense, this study answers the dual call in our field to include family dimensions in researchers’ conceptualizing and modeling, sampling and analyzing, and interpretations and implications (Aldrich and Cliff, 2003) and to further examine the role of context and institutions in future EI research (Fayolle and Liñán, 2014). Future research could follow suit in going beyond personal idiosyncrasies and their influence on individual evaluations of entrepreneurial activity (Dyer, 1995; Peterman and Kennedy, 2003; Timmons and Spinelli, 2003; Koe Hwee Nga and Shamuganathan, 2010; Santos et al., 2016) and examine our mediated model from new theoretical perspectives.

## Data Availability Statement

The raw data supporting the conclusions of this article will be made available by the authors, without undue reservation.

## Ethics Statement

The studies involving human participants were reviewed and approved by the Ethics Committee of Shanghai University of Finance and Economics. Written informed consent for participation was not required for this study in accordance with the national legislation and the institutional requirements.

## Author Contributions

XL, WZ, and HZ: conceptualization. XL: data curation, funding acquisition, investigation, and validation. XL and WZ: formal analysis, methodology, and writing–original draft. WZ: project administration. WZ and HZ: writing–review and editing. All authors contributed to the article and approved the submitted version.

## Conflict of Interest

The authors declare that the research was conducted in the absence of any commercial or financial relationships that could be construed as a potential conflict of interest.

## References

[B1] Ajzen I. (2001). Nature and operation of attitudes. *Ann. Rev. Psychol.* 52 27–58. 10.1146/annurev.psych.52.1.27 11148298

[B2] Aldrich H. E. Cliff J. E. (2003). The pervasive effects of family on entrepreneurship: toward a family embeddedness perspective. *J. Bus. Vent.* 18 573–596. 10.1016/S0883-9026(03)00011-9

[B3] Anderson R. E. Tatham R. L. Black W. C. Hair J. F. (1998). *Multivariate Data Analysis*, 5th Edn. Upper Saddle River, NJ: Prentice Hall College Div.

[B4] Armitage C. J. Conner M. (2001). Efficacy of the theory of planned behaviour: a meta-analytic review. *Br. J. Soc. Psychol.* 40 471–499. 10.1348/014466601164939 11795063

[B5] Atitsogbe K. A. Mama N. P. Sovet L. Pari P. Rossier J. (2019). Perceived employability and entrepreneurial intentions across university students and job seekers in togo: the effect of career adaptability and self-efficacy. *Front. Psychol.* 10:180. 10.3389/fpsyg.2019.00180 30800087 PMC6376950

[B6] Autio E. Robert H. K. Klofsten M. Parker G. G. C. Hay M. (2001). Entrepreneurial intent among students in scandinavia and in the USA. *Enter. Innov. Manag. Stud.* 2 145–160. 10.1080/14632440110094632

[B7] Azmat F. Fujimoto Y. (2016). Family embeddedness and entrepreneurship experience: a study of Indian migrant women entrepreneurs in Australia. *Entrepr. Reg. Dev.* 28 630–656. 10.1080/08985626.2016.1208279

[B8] Baluku M. M. Kikooma J. F. Otto K. König C. J. Bajwa N. ul. H (2020). Positive psychological attributes and entrepreneurial intention and action: the moderating role of perceived family support. *Front. Psychol.* 11:546745. 10.3389/fpsyg.2020.546745 33363491 PMC7753326

[B9] Bird B. (1988). Implementing entrepreneurial ideas: the case for intention. *Acad. Manag. Rev.* 13 442–453. 10.5465/amr.1988.4306970

[B10] Bourdieu P. Johnson R. (1993). *The Field of Cultural Production: Essays on art and Literature.* New York, NY: Columbia University Press.

[B11] Cardella G. M. Hernández-Sánchez B. R. Sánchez García J. C. (2020). Entrepreneurship and family role: a systematic review of a growing research. *Front. Psychol.* 10:2939. 10.3389/fpsyg.2019.02939 31998192 PMC6967397

[B12] Carr J. C. Sequeira J. M. (2007). Prior family business exposure as intergenerational influence and entrepreneurial intent: a theory of planned behavior approach. *J. Bus. Res.* 60 1090–1098. 10.1016/j.jbusres.2006.12.016

[B13] Cassar G. (2004). The financing of business start-ups. *J. Bus. Vent.* 19 261–283. 10.1016/S0883-9026(03)00029-6

[B14] Chandler G. N. Lyon D. W. (2001). Issues of research design and construct measurement in entrepreneurship research: the past decade. *Entrep. Theory Pract.* 25 101–113. 10.1177/104225870102500407

[B15] Chin W. W. (1998). “The partial least squares approach to structural equation modeling,” in *Modern Methods for Business Research*, ed. Marcoulides G. A. (Mahwah NJ: Lawrence Erlbaum Associates Inc), 295–336.

[B16] Cialdini R. B. (2005). Basic social influence is underestimated. *Psychol. Inquiry* 16 158–161. 10.1207/s15327965pli1604_03 26627889

[B17] Clough D. R. Fang T. P. Bala Vissa B. Wu A. (2019). Turning lead into gold: how do entrepreneurs mobilize resources to exploit opportunities? *Acad. Manag. Ann.* 13 240–271. 10.5465/annals.2016.0132

[B18] Coleman J. S. (1988). Social capital in the creation of human capital. *Am. J. Sociol.* 94 S95–S120. 10.1086/228943

[B19] Criaco G. Sieger P. Wennberg K. Chirico F. Minola T. (2017). Parents’ performance in entrepreneurship as a “double-edged sword” for the intergenerational transmission of entrepreneurship. *Small Bus. Econom.* 49 841–864. 10.1007/s11187-017-9854-x

[B20] Deutsch F. M. (2006). Filial piety, patrilineality, and China’s one-child policy. *J. Fam. Issu.* 27 366–389. 10.1177/0192513X05283097

[B21] Dyer W. G. (1995). Toward a theory of entrepreneurial careers. *Entrep. Theory Pract.* 19 7–21. 10.1177/104225879501900202

[B22] Dyer W. G. Nenque E. Hill E. J. (2014). Toward a theory of family capital and entrepreneurship: antecedents and outcomes. *J. Small Bus. Manag.* 2 266–285. 10.1111/jsbm.12097

[B23] Eesley C. Wang Y. (2017). Social influence in career choice: evidence from a randomized field experiment on entrepreneurial mentorship. *Res. Policy* 46 636–650. 10.1016/j.respol.2017.01.010

[B24] Fayolle A. Gailly B. Lassas−Clerc N. (2006). Assessing the impact of entrepreneurship education programmes: a new methodology. *J. Eur. Indust. Train.* 30 701–720. 10.1108/03090590610715022

[B25] Fayolle A. Liñán F. (2014). The future of research on entrepreneurial intentions. *J. Bus. Res.* 67 663–666. 10.1016/j.jbusres.2013.11.024

[B26] Feola R. Vesci M. Botti A. Parente R. (2019). The determinants of entrepreneurial intention of young researchers: combining the theory of planned behavior with the triple helix model. *J. Small Bus. Manag.* 57 1424–1443. 10.1111/jsbm.12361

[B27] Ga̧siorowski J. Rudowicz E. Safranow K. (2015). Motivation towards medical career choice and future career plans of Polish medical students. *Adv. Health Sci. Educ.* 20 709–725. 10.1007/s10459-014-9560-2 25352498 PMC4495256

[B28] Goethner M. Obschonka M. Silbereisen R. K. Cantner U. (2012). Scientists’ transition to academic entrepreneurship: economic and psychological determinants. *J. Econ. Psychol.* 33 628–641. 10.1016/j.joep.2011.12.002

[B29] Granovetter M. (1985). Economic-action and social-structure – the problem of embeddedness. *Am. J. Sociol.* 91 481–510. 10.1086/228311

[B30] Griffin B. Hu W. (2019). Parental career expectations: effect on medical students’ career attitudes over time. *Med. Educ.* 53 584–592. 10.1111/medu.13812 30734329

[B31] Grusec J. E. (2011). Socialization processes in the family: social and emotional development. *Ann. Rev. Psychol.* 62 243–269. 10.1146/annurev.psych.121208.131650 20731599

[B32] Hockerts K. (2017). Determinants of social entrepreneurial intentions. *Entrep. Theory Pract.* 41 105–130. 10.1111/etap.12171

[B33] Kan K. Tsai W.-D. (2006). Entrepreneurship and risk aversion. *Small Bus. Econom.* 26 465–474. 10.1007/s11187-005-5603-7

[B34] Karimi S. Makreet A. S. (2020). The role of personal values in forming students’ entrepreneurial intentions in developing countries. *Front. Psychol.* 11:525844. 10.3389/fpsyg.2020.525844 33329168 PMC7710526

[B35] Kautonen T. Tornikoski E. T. Kibler E. (2011). Entrepreneurial intentions in the third age: the impact of perceived age norms. *Small Bus. Econom.* 37 219–234. 10.1007/s11187-009-9238-y

[B36] Kautonen T. van Gelderen M. Fink M. (2015). Robustness of the theory of planned behavior in predicting entrepreneurial intentions and actions. *Entrepreneurship* 39 655–674. 10.1111/etap.12056

[B37] Kautonen T. van Gelderen M. Tornikoski E. T. (2013). Predicting entrepreneurial behaviour: a test of the theory of planned behaviour. *Appl. Econom.* 45 697–707. 10.1080/00036846.2011.610750

[B38] Kibler E. (2013). Formation of entrepreneurial intentions in a regional context. *Entrepr. Reg. Dev.* 25 293–323. 10.1080/08985626.2012.721008

[B39] Kickul J. Wilson F. Marlino D. Barbosa S. D. (2008). Are misalignments of perceptions and self-efficacy causing gender gaps in entrepreneurial intentions among our nation’s teens? *J. Small Bus. Enter. Dev.* 15 321–335. 10.1108/14626000810871709

[B40] Klein H. Kleinman D. (2002). The social construction of technology: structural considerations. *Sci. Technol. Hum. Values* 27 28–52. 10.1177/016224390202700102

[B41] Klein S. B. Astrachan J. H. Smyrnios K. X. (2005). The F-PEC scale of family influence: construction, validation, and further implication for theory. *Entrepreneurship* 29 321–339. 10.1111/j.1540-6520.2005.00086.x

[B42] Koe Hwee Nga J. Shamuganathan G. (2010). The influence of personality traits and demographic factors on social entrepreneurship start up intentions. *J. Bus. Ethics* 95 259–282. 10.1007/s10551-009-0358-8

[B43] Kreiser P. M. Marino L. D. Weaver K. M. (2002). Assessing the psychometric properties of the entrepreneurial orientation scale: a multi-country analysis. *Entrepr. Theory Pract.* 26 71–94. 10.1177/104225870202600405

[B44] Krueger N. Reilly M. D. Carsrud A. L. (2000). Competing models of entrepreneurial intentions. *J. Bus. Vent.* 15 411–432. 10.1016/S0883-9026(98)00033-0

[B45] Lefevre J. H. Roupret M. Kerneis S. Karila L. (2010). Career choices of medical students: a national survey of 1780 students. *Med. Educ.* 44 603–612. 10.1111/j.1365-2923.2010.03707.x 20604857

[B46] Lévesque M. Minniti M. (2006). The effect of aging on entrepreneurial behavior. *J. Bus. Vent.* 21 177–194. 10.1016/j.jbusvent.2005.04.003

[B47] Liñán F. Chen Y.-W. (2009). Development and cross-cultural application of a specific instrument to measure entrepreneurial intentions. *Entrep. Theory Pract.* 33 593–617. 10.1111/j.1540-6520.2009.00318.x

[B48] Liñán F. Nabi G. Kueger N. F. (2013). British and Spanish entrepreneurial intentions: a comparative study. *Rev. Econ. Mund.* 33 73–103. 10.1227/01.NEU.0000297044.82035.57

[B49] Liñán F. Rodríguez−Cohard J. C. (2015). Assessing the stability of graduates’ entrepreneurial intention and exploring its predictive capacity. *Acad. Rev. Latinoam. Admin.* 28 77–98. 10.1108/ARLA-06-2013-0071

[B50] Liñán F. Urbano D. Guerrero M. (2011). Regional variations in entrepreneurial cognitions: start-up intentions of university students in Spain. *Entrep. Reg. Dev.* 23 187–215. 10.1080/08985620903233929

[B51] Luis-Rico M.-I. Escolar-Llamazares M.-C. de la Torre-Cruz T. Herrero Á Jiménez A. Arranz Val P. (2020). The association of parental interest in entrepreneurship with the entrepreneurial interest of spanish youth. *Int. J. Environ. Res. Public Health* 17:4744. 10.3390/ijerph17134744 32630314 PMC7369814

[B52] Martin B. C. McNally J. J. Kay M. J. (2013). Examining the formation of human capital in entrepreneurship: a meta-analysis of entrepreneurship education outcomes. *J. Bus. Vent.* 28 211–224. 10.1016/j.jbusvent.2012.03.002

[B53] Masselink L. E. Lee S.-Y. D. Konrad T. R. (2008). Workplace relational factors and physicians’ intention to withdraw from practice. *Health Care Manag. Rev.* 33 178–187. 10.1097/01.HMR.0000304507.50674.28 18360168

[B54] Muñoz-Bullon F. Sanchez-Bueno M. J. Vos-Saz A. (2015). Startup team contributions and new firm creation: the role of founding team experience. *Entrep. Reg. Dev.* 27 80–105. 10.1080/08985626.2014.999719

[B55] Nunnally J. C. Bernstein I. H. (1994). *Psychometric Theory.* New York, NJ: McGraw-Hill.

[B56] Peterman N. E. Kennedy J. (2003). Enterprise education: influencing students’ perceptions of entrepreneurship. *Entrep. Theory Pract.* 28 129–144. 10.1046/j.1540-6520.2003.00035.x

[B57] Pinillos M.-J. Reyes L. (2011). Relationship between individualist–collectivist culture and entrepreneurial activity: evidence from Global Entrepreneurship Monitor data. *Small Bus. Econom.* 37 23–37. 10.1007/s11187-009-9230-6

[B58] Podsakoff P. M. MacKenzie S. B. Lee J.-Y. Podsakoff N. P. (2003). Common method biases in behavioral research: a critical review of the literature and recommended remedies. *J. Appl. Psychol.* 88 879–903. 10.1037/0021-9010.88.5.879 14516251

[B59] Podsakoff P. M. Organ D. W. (1986). Self-reports in organizational research: problems and prospects. *J. Manag.* 12 531–544. 10.1177/014920638601200408

[B60] Renzulli L. A. Aldrich H. Moody J. (2000). Family matters: gender, networks, and entrepreneurial outcomes. *Soc. Forc.* 79 523–546. 10.1093/sf/79.2.523 32080734

[B61] Roggenkamp S. D. White K. R. (1998). Four nurse entrepreneurs: what motivated them to start their own businesses. *Health Care Manag. Rev.* 23 67–75. 10.1097/00004010-199807000-00008 9702563

[B62] Santos F. J. Roomi M. A. Liñán F. (2016). About gender differences and the social environment in the development of entrepreneurial intentions. *J. Small Bus. Manag.* 54 49–66. 10.1111/jsbm.12129

[B63] Schmutzler J. Andonova V. Diaz-Serrano L. (2019). How context shapes entrepreneurial self-efficacy as a driver of entrepreneurial intentions: a multilevel approach. *Entrepr. Theory Pract.* 43 880–920. 10.1177/1042258717753142

[B64] Shane S. (2003). *A General Theory of Entrepreneurship: The Individual-opportunity Nexus.* Cheltenham: Edward Elgar Publishing. 10.4337/9781781007990

[B65] She L. Wu B. Xu L. Wu J. Zhang P. Li E. (2008). Determinants of career aspirations of medical students in southern China. *BMC Med. Educ.* 8:59. 10.1186/1472-6920-8-59 19077214 PMC2621218

[B66] Souitaris V. Zerbinati S. Al-Laham A. (2007). Do entrepreneurship programmes raise entrepreneurial intention of science and engineering students? The effect of learning, inspiration and resources. *J. Bus. Vent.* 22 566–591. 10.1016/j.jbusvent.2006.05.002

[B67] Steier L. (2003). Variants of agency contracts in family-financed ventures as a continuum of familial altruistic and market rationalities. *J. Bus. Vent.* 18 597–618. 10.1016/S0883-9026(03)00012-0

[B68] Steier L. (2007). New venture creation and organization: a familial sub-narrative. *J. Bus. Res.* 60 1099–1107. 10.1016/j.jbusres.2006.12.017

[B69] Sullivan R. (2000). Entrepreneurial learning and mentoring. *Int. J. Entrep. Behav. Res.* 6 160–175. 10.1108/13552550010346587

[B70] Timmons J. A. Spinelli S. (2003). *New Venture Creation: Entrepreneurship for the 21st Century.* New York, NY: McGraw-Hill.

[B71] Tkachev A. Kolvereid L. (1999). Self-employment intentions among Russian students. *Entrep. Reg. Dev.* 11 269–280. 10.1080/089856299283209

[B72] Urman R. D. Ehrenfeld J. M. (2011). “Physicians’ pathways to non-traditional careers and leadership opportunities,” in *Physicians’ Pathways to Non-Traditional Careers and Leadership Opportunities*, Vol. 9781461405 eds Urman R. D. Ehrenfeld J. M. (Berlin: Springer). 10.1007/978-1-4614-0551-1

[B73] van Gelderen M. Brand M. van Praag M. Bodewes W. Poutsma E. van Gils A. (2008). Explaining entrepreneurial intentions by means of the theory of planned behaviour. *Career Dev. Int.* 13 538–559. 10.1108/13620430810901688

[B74] Veciana J. M. Aponte M. Urbano D. (2005). University students’ attitudes towards entrepreneurship: a two countries comparison. *Int. Entrep. Manag. J.* 1 165–182. 10.1007/s11365-005-1127-5

[B75] Wang S.-M. Yueh H.-P. Wen P.-C. (2019). How the new type of entrepreneurship education complements the traditional one in developing entrepreneurial competencies and intention. *Front. Psychol.* 10:2048. 10.3389/fpsyg.2019.02048 31572260 PMC6753869

[B76] West S. G. Finch J. F. Curran P. J. (1995). “Structural equation models with non-normal variables: Problems and remedies,” in *Structural Equation Modeling: Concepts, Issues, and Applications*, ed. Hoyle R. H. (Thousand Oaks, CA: Sage Publications), 56–75.

[B77] Wu S. Wu L. (2008). The impact of higher education on entrepreneurial intentions of university students in China. *J. Small Bus. Enter. Dev.* 15 752–774. 10.1108/14626000810917843

[B78] Yeh K.-H. Yi C.-C. Tsao W.-C. Wan P.-S. (2013). Filial piety in contemporary Chinese societies: a comparative study of Taiwan, Hong Kong, and China. *Int. Sociol.* 28 277–296. 10.1177/0268580913484345

[B79] Zellweger T. Sieger P. Halter F. (2011). Should I stay or should I go? Career choice intentions of students with family business background. *J. Bus. Vent.* 26 521–536. 10.1016/j.jbusvent.2010.04.001

[B80] Zhao J. Wei G. Chen K.-H. Yien J.-M. (2020). Psychological Capital and university students’ entrepreneurial intention in china: mediation effect of entrepreneurial capitals. *Front. Psychol.* 10:2984. 10.3389/fpsyg.2019.02984 32038375 PMC6989491

